# Upregulation of the ZNF148/PTX3 axis promotes malignant transformation of dendritic cells in glioma stem‐like cells microenvironment

**DOI:** 10.1111/cns.14213

**Published:** 2023-04-17

**Authors:** Shan Cheng, Liang Liu, Delin Wang, Yongdong Li, Suwen Li, Jiaqi Yuan, Shilu Huang, Zhipeng Xu, Bin Jia, Zhe Li, Jun Dong

**Affiliations:** ^1^ Department of Neurosurgery The Second Affiliated Hospital of Soochow University Suzhou Jiangsu China; ^2^ Department of Neurosurgery Jiande First People's Hospital Hangzhou Zhejiang China; ^3^ State Key Laboratory of Analytical Chemistry for Life Science Nanjing University Nanjing Jiangsu China

**Keywords:** dendritic cells, GBM microenvironment, glioma stem cells, malignant transformation, transcription factor ZNF148

## Abstract

**Background:**

The recent development of dendritic cell (DC)‐based immunotherapy has resulted in advances in glioblastoma multiforme (GBM) treatment. However, the cell fate of DCs in the GBM microenvironment, especially in microenvironments in which glioma stem cell (GSCs)‐mediated remodeling has resulted in highly immunosuppressive conditions, has not yet been fully investigated.

**Methods:**

Observed the interaction between GSCs and primary cultured DCs in a dual‐color tracing model, monoclonal and continuously passaged highly proliferative DCs, and named transformed DCs (t‐DCs). The expression of DC‐specific surface markers was analyzed using RT‐PCR, chromosome karyotype, and flow cytometry. The expression of long pentraxin 3 (PTX3) and its transcription factor zinc finger protein 148 (ZNF148) in t‐DCs was detected using qRT‐PCR and western blot. CCK8 and transwell assays were conducted to assess the effect of ZNF148 and PTX3 on the proliferation, migration, and invasion of t‐DCs. Bioinformatics analysis, dual‐luciferase reporter assay, and chromatin immunoprecipitation (ChIP)‐qPCR assay were used to explore the relation between ZNF148 and PTX3.

**Results:**

Transformed DCs (t‐DCs) still expressed DC‐specific surface markers, namely, CD80 and CD11c, and immune‐related costimulatory molecules, namely, CD80, CD86, CD40, and ICAM‐1. However, the expression levels of these molecules in t‐DCs decreased moderately compared to those in naive DCs. Stable overexpression of PTX3 further promoted the proliferation and migration of t‐DCs in vitro, decreased the expression of costimulatory molecules, and increased the tumorigenicity of t‐DCs in vivo. The transcription factor zinc finger protein 148 (ZNF148) was directly bound to the PTX3 promoter region and enhanced PTX3 expression. Downregulation of ZNF148 significantly decreased PTX3 expression and reduced the proliferation and migration of t‐DCs. Overexpression of ZNF148 significantly increased PTX3 expression and promoted the proliferation and migration of t‐DCs, achieving the same biological effects as PTX3 overexpression in t‐DCs. Simultaneously, the downregulation of ZNF148 partially reversed the effect of PTX3 overexpression in t‐DCs.

**Conclusion:**

The ZNF148/PTX3 axis played an important role in regulating the malignant transformation of DCs after cross‐talk with GSCs, and this axis may serve as a new target for sensitizing GBM to DC‐based immunotherapy.

## Graphical abstract

The transcription factor ZNF148 promotes the malignant transformation of dendritic cells after cross‐talk with glioma stem cells by upregulating PTX3, and reduce the expression of co‐stimulatory factors on the surface of DCs, inhibit their antigen presentation and activation of T cells.

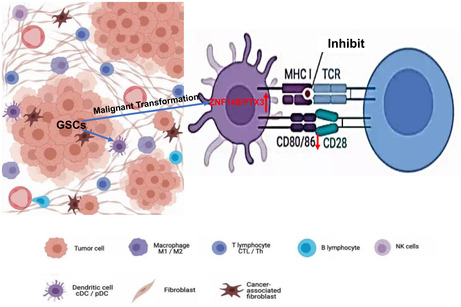

## 1 INTRODUCTION

Glioblastoma multiforme (GBM), which is the most common primary intracranial malignancy in adults, has always been a great challenge in the field of neuro‐oncology.^1^ Although certain progressions have been made against GBM in recent years, patient outcomes remain poor due to uncontrollable recurrence and rapid progression.^2^ Therefore, further elucidation of unidentified tumorigenesis mechanisms to explore new potential therapeutic strategies is under urgent desire. Accumulating data have shown that the refractory nature of GBM is inseparable from glioma stem cells (GSCs)‐mediated tissue remodeling of tumor microenvironment (TME). Direct interactions between tumor stromal cells and GSCs, and indirect interactions via GSCs‐derived small molecules, contribute greatly to GBM development and progression.^3^

GSCs are also reported as the core to initiate remodeling of GBM immune‐microenvironment; however, the mutual interactions between GSCs and antigen‐presenting dendritic cells (DCs), as well as the cell fate of DCs in the highly immuno‐suppressive GBM microenvironment have not been fully elucidated. During the processes of GBM development, DCs can reach brain tissue via afferent lymphatic vessels or endothelial venules,^4^ which constitute the basis of antitumor immunity.^5^, ^6^, ^7^, ^8^ However, the specific roles of DCs in GBM development are complex and have not been fully elucidated. Recent studies have reported that DCs with increased phagocytosis were present within glioma TME.^9^, ^10^ DC vaccines have already been applied in immunotherapy against many non‐CNS malignancies and achieved obvious therapeutic efficacy.^11^, ^12^ These advances have inspired great interest in clinical immunotherapeutic trials of DC‐based vaccines against GBM.^13^ The latest study has reported that DC‐based immunotherapy has achieved positive results related to survival benefits,^14^ which can be considered one of the great breakthroughs in GBM therapy. They have further prolonged the OS of GBM patients by at least 6 months, which can be compared with the other two effective therapies, namely, tumor‐treating fields and TMZ. However, it is still far from curing GBM (the most refractory CNS malignancy), which deserves further preclinical investigations to continuously improve the practical efficacy of DCs‐based target therapy.^14^, ^15^ The significant local and systemic immunosuppressive TME remodeled by GSCs may limit clinical efficacy of the modified DC vaccines, supporting the necessity of exploring new strategy of targeting transformed DCs (t‐DCs) to improve patients' prognosis, which should be based on clarifying the exact mechanisms of mutual interactions between GSCs and DCs.

Our previous studies have already observed that GSCs can induce the malignant transformation of several types of TME stromal cells,^16^, ^17^, ^18^, ^19^, ^20^ including DCs.^21^, ^22^ However, the potential molecular mechanisms of DCs' transformation have not been investigated. Previous studies have shown that tumor‐derived IL‐6, TGF‐β1, IL‐10, and VEGF can induce DCs transformation to acquire a tolerogenic phenotype that is characterized by the low expression of costimulatory molecules.^23^, ^24^ Besides, herpes simplex virus infection and mitomycin C17 treatment can reduce costimulatory molecule expression in DCs, and both can convert DCs into resistant cells, resulting in the inhibition of T‐cell activation. Such tolerant DCs expressing low costimulatory molecule levels can inhibit intertumoral antigen‐specific CD8+ CTL activation.^25^, ^26^ However, whether GSCs can induce tolerant DCs to support GBM tissue remodeling has not been investigated previously.

The expression of the long pentraxin 3 (PTX3) in DCs is related to the humoral immune function of DCs, as a part of soluble pattern recognition molecules, participates in the recognition of pathogenic microorganisms, as well as activation of the immune system.^27^ In addition, the expression level of PTX3 was positively correlated with tumor malignancy degree,^28^, ^29^ sustained the migration and proliferation of tumor cells, and dysregulates mitogenic signaling pathways, encouraging tumor escape from immune responses.^30^ Our investigations disclosed that t‐DCs in the GSCs microenvironment exhibited obvious upregulation of PTX3, indicating the potential role of PTX3 in DC malignant transformation induced by GSCs.

## 2 MATERIALS AND METHODS

### 2.1 Animals and cells

Transgenic EGFP‐BALB/c nude mice were established and bred in our laboratory as previously described.^16^ All the animal experimental methods and protocols were performed in strict accordance with the guidelines that were approved by the Research Ethics Committee and Animal Care Committee of Soochow University (Approval No. SUDA20210708A03). RFP‐expressing SU3 human GSCs (SU3‐RFP cells) were previously derived from surgical GBM specimens in our laboratory,^16^, ^17^ and SU3 cells were positive for CD133 and nestin, which were consistent with the characteristics of GSCs.^31^, ^32^ These cells were cultured in Dulbecco's modified Eagle's medium/F12 medium (DMEM/F12 medium, Gibco) supplemented with 20 ng/mL basic fibroblast growth factor (bFGF; Gibco) and 20 ng/mL epidermal growth factor (EGF; Gibco).

### 2.2 Primary DC culture

The femurs and tibias were harvested from EGFP‐BALB/c nude mice under general anesthesia. Then, the bone marrow cells were removed by flushing the bones, washed with phosphate‐buffered saline (PBS), and treated with erythrocyte lysis buffer at 37°C for 5 min. The cells were suspended and cultured in RPMI 1640 culture medium supplemented with 10% fetal bovine serum (FBS, Gibco), 10 ng/mL recombinant murine granulocyte macrophage‐colony stimulating factor (GM‐CSF; R&D Systems) and 5 ng/mL recombinant murine interleukin‐4 (rmIL‐4; PeproTech). Half of the culture medium was replaced every day. On day 5, 1 μg/mL lipopolysaccharide (LPS; Sigma) was added to stimulate DC maturation. After 7 days, mature DCs were enriched and collected for further experiments.

### 2.3 Coculture of DCs and SU3‐RFP cells

EGFP^+^ DCs and SU3‐RFP cells were digested and resuspended into single‐cell suspensions. Then, the cells were mixed at a ratio of 10:1, seeded in poly‐L‐lysine solution‐coated six‐well plates, and cocultured in a humidified incubator at 37°C in 5% CO_2_. The interactions between the EGFP^+^ DCs and RFP^+^ SU3 cells were observed with a live‐cell imaging system with a fluorescence microscope.

### 2.4 Cloning of highly proliferative EGFP ^+^ DCs

After being cocultured for 10 days, the cocultured cell populations were digested into single‐cell suspensions, and the EGFP^+^ cells were harvested via fluorescence‐activated cell sorting. Highly proliferative EGFP^+^ cells were monoclonal with micropipette techniques under fluorescence microscopy, further cultured in a 96‐well plate, and named t‐DCs.

### 2.5 Polymerase chain reaction (PCR) and quantitative real‐time PCR

PCR was performed to measure the expression levels of ZNF148, PTX3, the DC markers CD11c, CD80, and SIRPα, and the macrophage markers F4/80 in t‐DCs.^33^, ^34^, ^35^ T‐DCs in the logarithmic growth phase were collected, and cell total RNA was extracted using TRIzol (Invitrogen). After measuring the concentration and performing the quality control step, total RNA was reverse transcribed into cDNA with a reverse transcription cDNA Kit (Thermo Fisher). Next, qRT–PCR was conducted with a Real‐Time PCR Kit (Takara) and the ABI 7500 system (Applied Biosystems).

The relative mRNA expression of target genes was calculated by the 2^−ΔΔct^ method. GAPDH was used as the endogenous control to normalize the data. The primer sequences are listed in Table 1.

**TABLE 1:** Primers used for PCR and qRT‐PCR analysis.

Primer name	Primer sequence (5′–3′)
EGFP	F: GCCACAAGTTCAGCGTGTCCG R: GTTGGGGTCTTTGCTCAGGGCG
CD80	F: CAACTGTCCAAGTCAGTGAAAG R: CACCACTTTGTCATGTTTTTGC
CD11c	F: TCATCACTGATGGGAGAAAACA R: CCCCAATTGCATAACGAATGAT
F4/80	F: CAGCTGTCTTAGAGGCTTCTCTT R: TGTAGCTTCCCACAGACTTAGAG
SIRPα	F: CTGTTGATCTACAGTTTCGCAG R: GGGTGACATTACTGATACGGAT
β‐actin(H)	F: ACATCCGCAAAGACCTGTAC R: GCCATGCCAATCTCATCTTG
β‐actin(M)	F: CTTTGCAGCTCCTTCGTTG R: TGGTAACAATGCCATGTTCA
ZNF148	F: ACTCGTCGAGCACTAAAGTAA R: TTTTTGAGAACCAACTTGGGTG
PTX3	F: TCGAAGAAGATTTTTGGAAGCG R: CTGAGGTACAGCTGAATCTCAT

### 2.6 Flow cytometry

To analyze cell surface marker expression, DCs or t‐DCs (2 × 10^6^) were harvested, and FITC‐conjugated anti‐CD11c, anti‐CD80, anti‐CD86, anti‐CD40, and anti‐ICAM‐1 (1:200, Biolegend) antibodies were incubated with the cells in a total volume of 100 μL for 30 min at room temperature. Then, the cells were washed twice with 1 mL PBS buffer, centrifuged at 179 *g* for 5 min, resuspended in 500 μL PBS, and analyzed with a flow cytometer (BD, USA). Then, the data were analyzed with FlowJo software. Isotype controls were used as the appropriate controls to determine the median fluorescence intensity by flow cytometry.

To analyze cell cycle progression, cells were washed with PBS three times and fixed with 75% ice‐cold ethanol. Then, the cells were incubated in PBS with propidium iodide (PI; 25 μg/mL) and 10 mg/mL RNase for 30 min in the dark before being analyzed by flow cytometry.

To assess cell apoptosis, the proportion of Annexin V1‐positive cells was determined using an Annexin V‐FITC (fluorescein isothiocyanate) apoptosis detection kit (BD Pharmingen) according to the manufacturer's instructions. Briefly, a total of 1 × 10^5^ cells were resuspended in binding buffer, the cells were labeled with 5 μL FITC‐conjugated Annexin V and 5 μL PI without permeabilization, then the cells were subsequently analyzed by flow cytometry with FlowJo software. The proportion of early apoptotic cells located in the lower right quadrant was considered the proportion of apoptotic cells.

### 2.7 Cell counting kit‐8 (CCK‐8) assay

Cells were seeded in a 96‐well plate at a density of 800 cells/well in 100 μL DMEM. Blank controls (treated with only medium and CCK‐8 reagent) were established. Ten microliters of CCK8 reagent (Dojindo) was added to each well every 24 h, then the cells were incubated in an incubator for another 2 h at 37°C. The absorbance value was recorded at 450 nm with a spectrophotometer (Tecan).

### 2.8 Colony formation assay

Cells were collected following trypsin–EDTA digestion and diluted to generate a low‐density single‐cell suspension (1 × 10^4^ cells/mL). Thirty microliters of this suspension was added to each well of a six‐well plate. After 2 weeks of culture, the colonies that had formed in the six‐well plate were counted, and colony formation rates were calculated.

### 2.9 Migration and invasion assay

Transwell assay was performed to evaluate the migration and invasion of t‐DCs. The invasion and migration assays differed according to whether the upper chambers were coated with Matrigel or not (50 μL, 1:8 dilution, BD, USA). Chamber inserts (Merck Millipore) were precoated with 100 μL of Matrigel. For both the migration and invasion assays, 5 × 10^4^ cells in serum‐free medium were seeded into the upper chamber, and DMEM supplemented with 10% FBS was added to the lower chamber. After incubation for 48 h at 37°C in 5% CO_2_ in an incubator, the cells in the upper chamber were scraped and washed away. Additionally, the cells in the lower chambers were fixed using 4% paraformaldehyde for 5 min, stained with 1% crystal violet for 30 min, and washed with PBS three times. The cells that had migrated or invaded were observed and counted in at least three randomly selected fields under an optical microscope, and the results were analyzed with ImageJ software.

### 2.10 Wound healing assay

Cells were seeded in a six‐well plate and cultured in DMEM supplemented with FBS at 37°C overnight. A 200 μL pipette tip was used to make wounds in the cell monolayer. Then, the cells were washed with PBS and cultured in DMEM without FBS. Twenty‐four hours later, the cells were observed under an inverted microscope (AMG, USA) at 10× magnification. Images of the wounded area were captured and analyzed via ImageJ software.

### 2.11 Western blotting analysis

Total cellular proteins were extracted using RIPA buffer (KenGEN). The protein concentration was determined with a BCA Protein Assay Kit (Beyotime). Next, total proteins were separated by 10% SDS–PAGE. Then, the separated proteins were transferred to PVDF membranes (Millipore), which were then blocked for 2 h at room temperature with 5% skim milk. The membranes were incubated with the following primary antibodies at 1:500 dilutions overnight at 4°C: anti‐ZNF148 (A7001, Abclonal) or anti‐PTX3 (12669, Abclonal). After washing with TBST three times, the PVDF membranes were incubated with horseradish peroxidase‐conjugated secondary antibody (1:5000, GAM0072, MULTI SCIENCES). GAPDH (1:20,000, 10494‐1‐AP, Proteintech) was used as the control. The bands on the PVDF membranes were visualized and images were captured under Tanon 4600SF (Tanon).

### 2.12 Plasmid construction and transfection

Small interfering RNA (siRNA) to knock down ZNF148 expression, plasmids for ZNF148 or PTX3 overexpression, and the corresponding negative controls and vectors (Genepharma) were constructed for this experiment. Cells were transfected with these constructs using Lipofectamine 3000 (Invitrogen) according to the manufacturer's instructions.

### 2.13 Chromatin immunoprecipitation (ChIP)‐ qPCR assay

ChIP assay was performed using BeyoCHIPTM CHIP Assay Kit (Beyotime) according to the manufacturer's instructions. T‐DCs were fixed via the addition of formaldehyde (final concentration of 1%) for 10 min, then administering glycine was to neutralize extra formaldehyde. Cells were washed with PBS, lysed with SDS Lysis Buffer for 10 min, then sonicated under 4°C to shear the cross‐linked DNA to 200–1000 bp. The cross‐linked protein‐DNA was immunoprecipitated with magnetic beads, and protein‐DNA complexes were precipitated using IgG and anti‐ZNF148 antibody (Abclonal) overnight. After that, the eluted DNA fragments were detected with PTX3 promoter‐specific primers and SYBR premix.

### 2.14 Dual‐luciferase reporter assay

T‐DCs were cotransfected with pCDNA3.1‐ZNF148, Renilla Fluorescein TK, and either the full‐length sequence or three truncated fragments (Δ1, Δ2, or Δ3) of the PTX3 promoter region, which was inserted into the pGL3 vector. Forty‐eight hours posttransfection, the cells were harvested and lysed using the Dual‐Luciferase Reporter Assay System (Promega). The fluorescence intensity of cell lysates was measured using an imaging microplate reader.

### 2.15 Tumorigenicity assay

Athymic BALB/c nude mice (4 weeks old, 15–20 g) were bred in the SPF experimental animal center. Then, 1 × 10^6^ t‐DCs in which ZNF148 or PTX3 was up‐ or downregulated as well as the corresponding negative control cells were subcutaneously injected into the right flanks of mice. After 5 weeks, all the mice were sacrificed under general anesthesia to harvest the subcutaneous tumors, and the volume of the subcutaneous tumors was quantified (volume was calculated according to the following formula: *V* (mm^3^) = length × width^2^ × 0.5).

### 2.16 Isolation of exosomes

SU3 cells were maintained in the neurosphere culture medium favored for GSCs growth for 48 h (until 80%–90% confluence) as described previously, then the culture medium was collected and centrifuged at 2500 *g* for 20 min to remove cell debris. The supernatant was filtered with a 0.22 μm steriflip (Millipore), followed by concentration in a 50‐kDa ultracentrifuge tube (Amicon Ultra 15; Millipore) at 5000 *g* for 15 min. Exosomes of SU3 cells (SU3exo) were subsequently isolated with the exosome purification kit ExoQuick‐TC™ (System Bioscience) following the manufacturer's instructions and stored at −80°C for subsequent analysis.

### 2.17 Statistical analysis

All statistical analyses were conducted with GraphPad Prism 9.0. Results are presented as means ± SD based on three independent experiments. The normality of the data distribution was analyzed by the Shapiro–Wilk test. Statistical comparisons between two groups were conducted by the two‐tailed Student's test, and comparisons between more than two groups were analyzed by analysis of variance (ANOVA). A *p* < 0.05 (*) was considered statistically significant.

## 3 RESULTS

### 3.1 Primary culture of GFP+ DCs

DCs were isolated from the bone marrow of GFP‐BALB/c nude mice. For the primary culture of DCs, during the first 3 days, most of the cells were round in shape and grew in clusters. After 7 days, the primary cultured cells exhibited a stellate morphology and typical characteristics of dendritic‐like cells (Figure 2A), and flow cytometry showed that the proportion of cells that expressed the DC surface markers CD80 and CD11c were 86.5% and 79.4%, respectively (Figure 2B).

### 3.2 Coculture of GSCs with DCs in a dual‐color tracing model and cloning of t‐DCs

**FIGURE 1:**
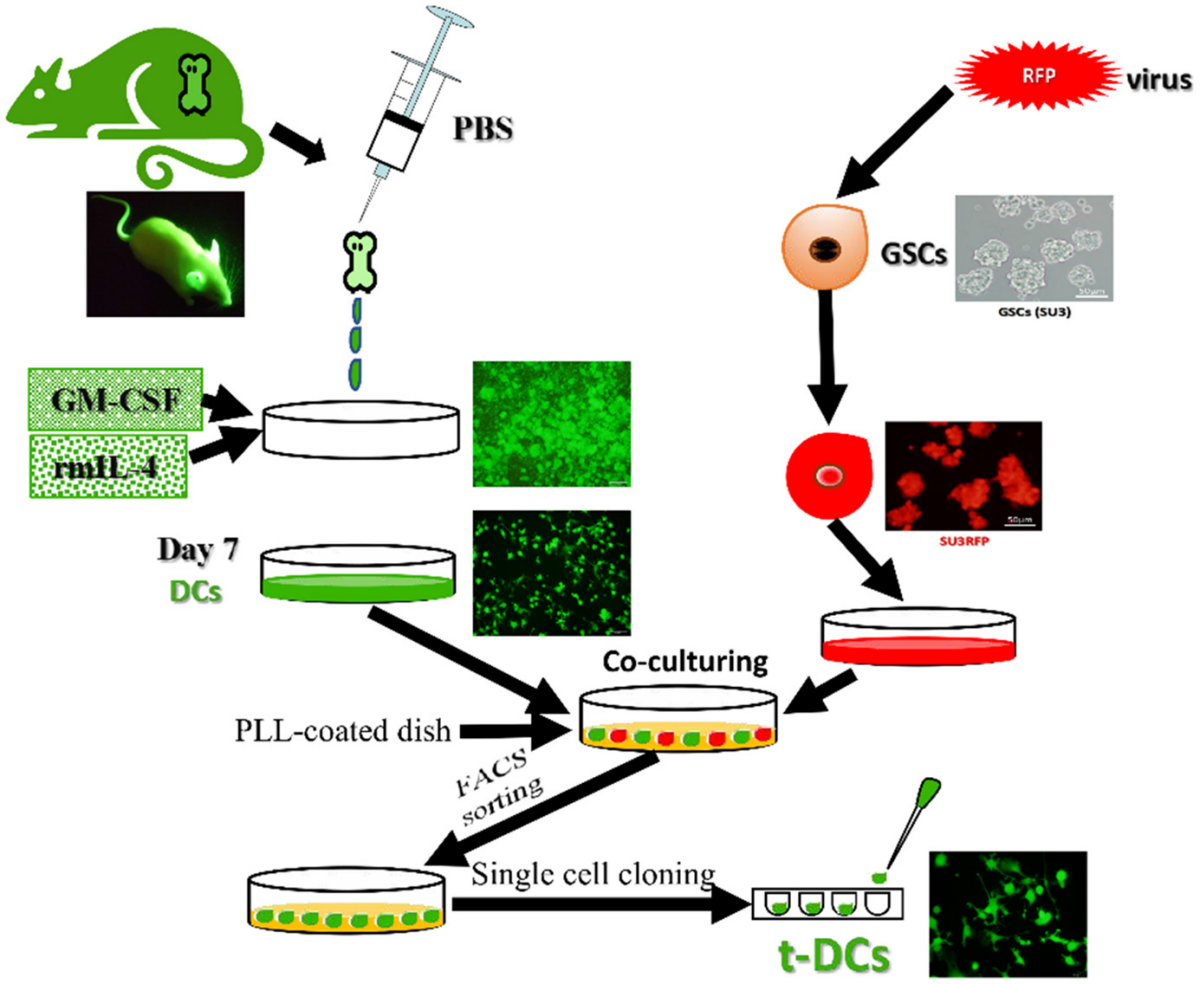
Schematic diagram of the construction of in vivo mutual interaction model of EGFP+ DCs(t‐DCs) versus RFP^+^ GSCs in dual fluorescence tracing platform.

**FIGURE 2:**
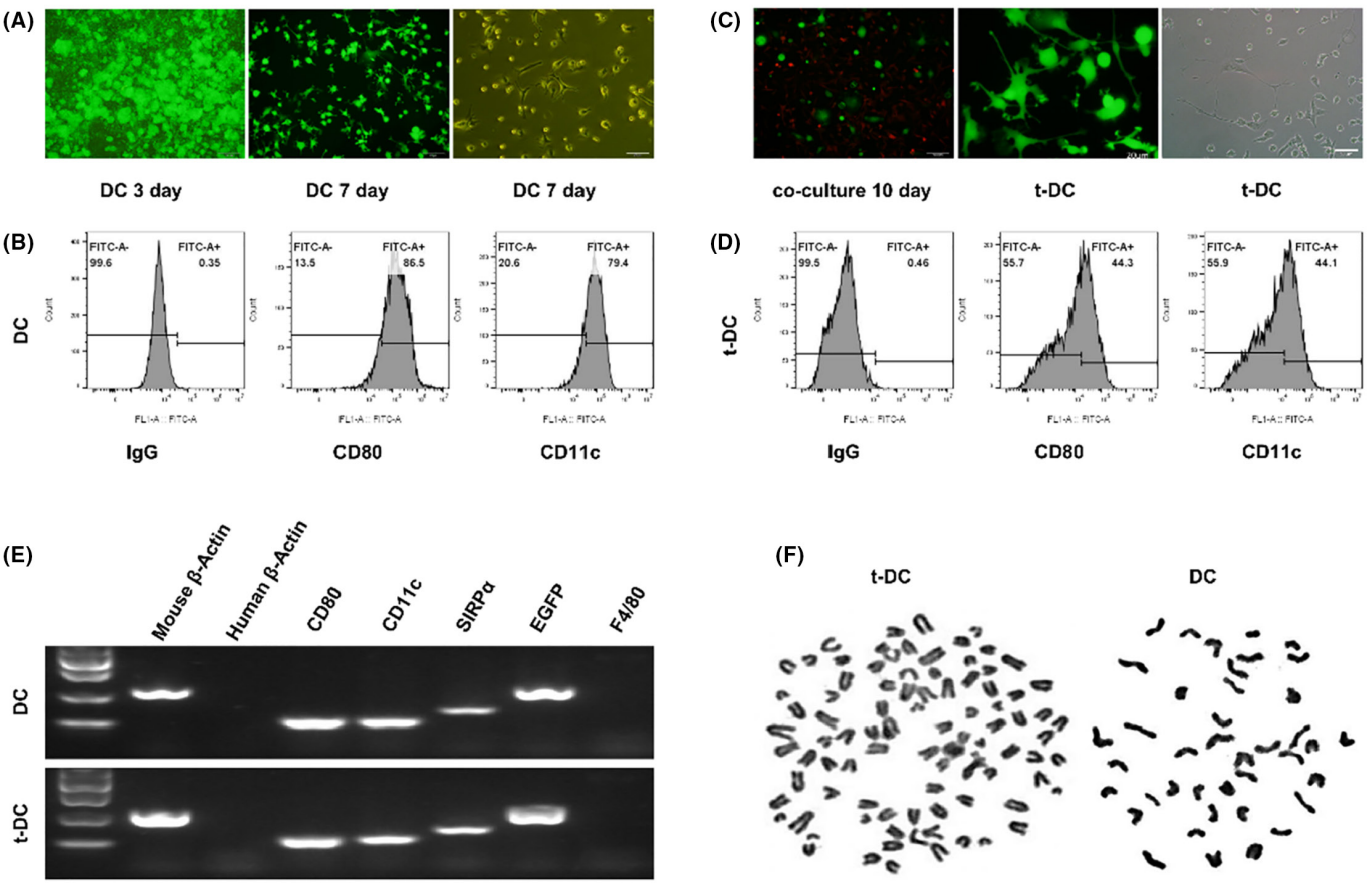
Comparison of phenotypes of t‐DCs and normal DCs. (A) Primary culture of DCs from GFP BALB/c nude mice. (B) Flow cytometry for CD80 and CD11c expression of DCs. (C) Cloning of highly proliferative EGFP+ DCs. (D) Flow cytometry for CD80 and CD11c expression of t‐DCs. (E) RT‐PCR analysis for cell surface markers of both normal DCs and t‐DCs. (F) The chromosome karyotype of DCs and t‐DCs.

The human SU3 GSC cell line was stably transfected with the RFP gene. Then, GFP^+^ DCs were cocultured with RFP^+^ SU3 cells at a ratio of 10:1. The experimental process is shown in Figure 1. After being cocultured for 10 days, some highly proliferative EGFP^+^ DCs were monoclonal with micropipette techniques (Figure 2C) and then further cultured and passaged continuously. The flow cytometry results showed that the proportions of t‐DCs that expressed CD80 and CD11c were 44.3% and 44.1% (about half percent of naive DCs), respectively (Figure 2D).

RT–PCR analysis of the expression of the cell surface markers CD80, CD11c, SIRP‐α, and F4/80 showed that similar to normal DCs, t‐DCs expressed CD80, CD11c, and SIRP‐α, but they did not express F4/80 (Figure 2E). Chromosome karyotype analysis showed that the chromosomes of both DCs and t‐DCs were telo chromosomes and morphologically consistent with murine chromosomes; the DCs were normal and diploid, while the t‐DCs were heteroploid (Figure 2F).

### 3.3 Analysis of the proliferation and invasion of t‐DCs

**FIGURE 3:**
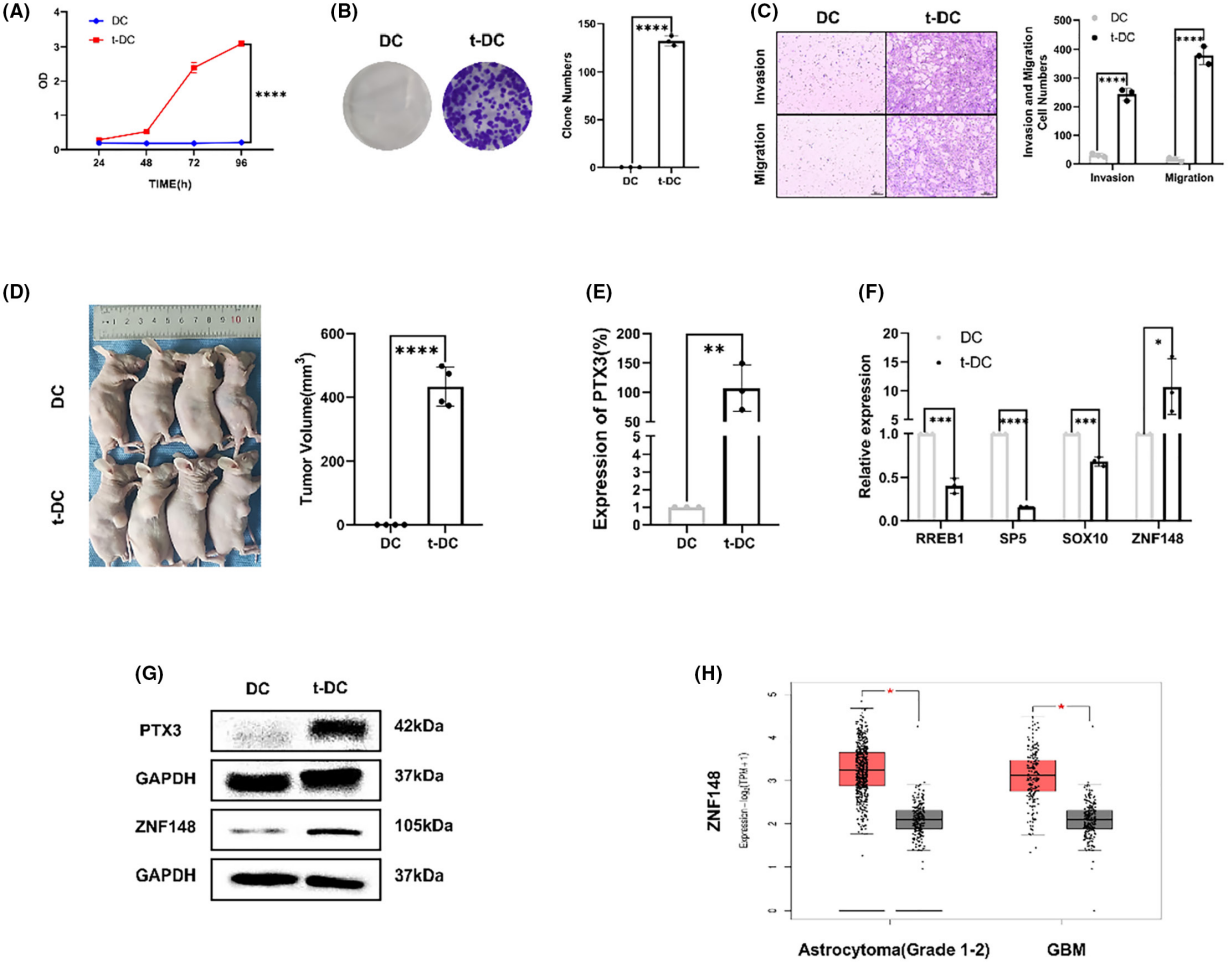
Analysis of the proliferation and invasion of t‐DCs. (A, B) Analysis of cell proliferation of DCs and t‐DCs by CC8 and clone formation assay. (C) Cell invasion and migration assay of t‐DCs. (D) Tumorigenicity assay of t‐DCs. (E) Quantitative RT‐PCR on PTX3 expression in t‐DCs. (F) Quantitative RT‐PCR on 4 candidate TFs, including RREB1, SP5, SOX10, and ZNF148 expression in t‐DCs. (G) Western blot on PTX3 and ZNF148 expression in t‐DCs. (H) ZNF148 expression of gliomas in TCGA database. Data are expressed as mean ± SD, *p < 0.05, **p < 0.01, ***p < 0.001.

The CCK8 assay revealed that the t‐DCs acquired the ability of infinite proliferation, they acquired an immortal phenotype (Figure 3A). The clonogenic assay showed that t‐DCs exhibited high clonogenicity (Figure 3B). The invasion and migration experiments proved that t‐DCs exhibited strong invasion and migration abilities (Figure 3C). The tumorigenicity experiment verified that subcutaneous transplantation of 1 × 10^6^ t‐DCs led to 100% tumor formation, indicating that t‐DCs acquired definite tumorigenicity in vivo (Figure 3D).

### 3.4 Proliferation‐related gene pentraxin 3 (PTX3) upregulation in t‐DCs

According to the results of differential gene expression analysis compared with naive DCs, qRT–PCR and western blotting revealed PTX3 upregulation in t‐DCs at both the transcriptional and protein levels (Figure 3E,G). Besides, databases were applied with online bioinformatic analysis (JASPAR and UCSC) to predict the transcription factors that regulate PTX3 expression. The selection criterion was set as: the transcription factors that regulate the transcription direction should be consistent with PTX3, and the correlation score is greater than 600, which resulted in four candidate TFs, including RREB1, SP5, SOX10, and ZNF148. The result is shown in Figure S1. qRT–PCR was performed to evaluate four candidate TFs expressions in both t‐DCs and DCs, which disclosed only ZNF148 upregulation in t‐DCs (Figure 3F), and was further verified by western blotting (Figure 3G). ZNF148 was also highly expressed in astrocytoma (Grade 1–2) and GBM samples according to the cancer genome atlas (TCGA) database (Figure 3H).

### 3.5 Knockdown or upregulation of the transcription factor ZNF148 suppressed or promoted the malignant phenotype of t‐DCs, respectively

**FIGURE 4:**
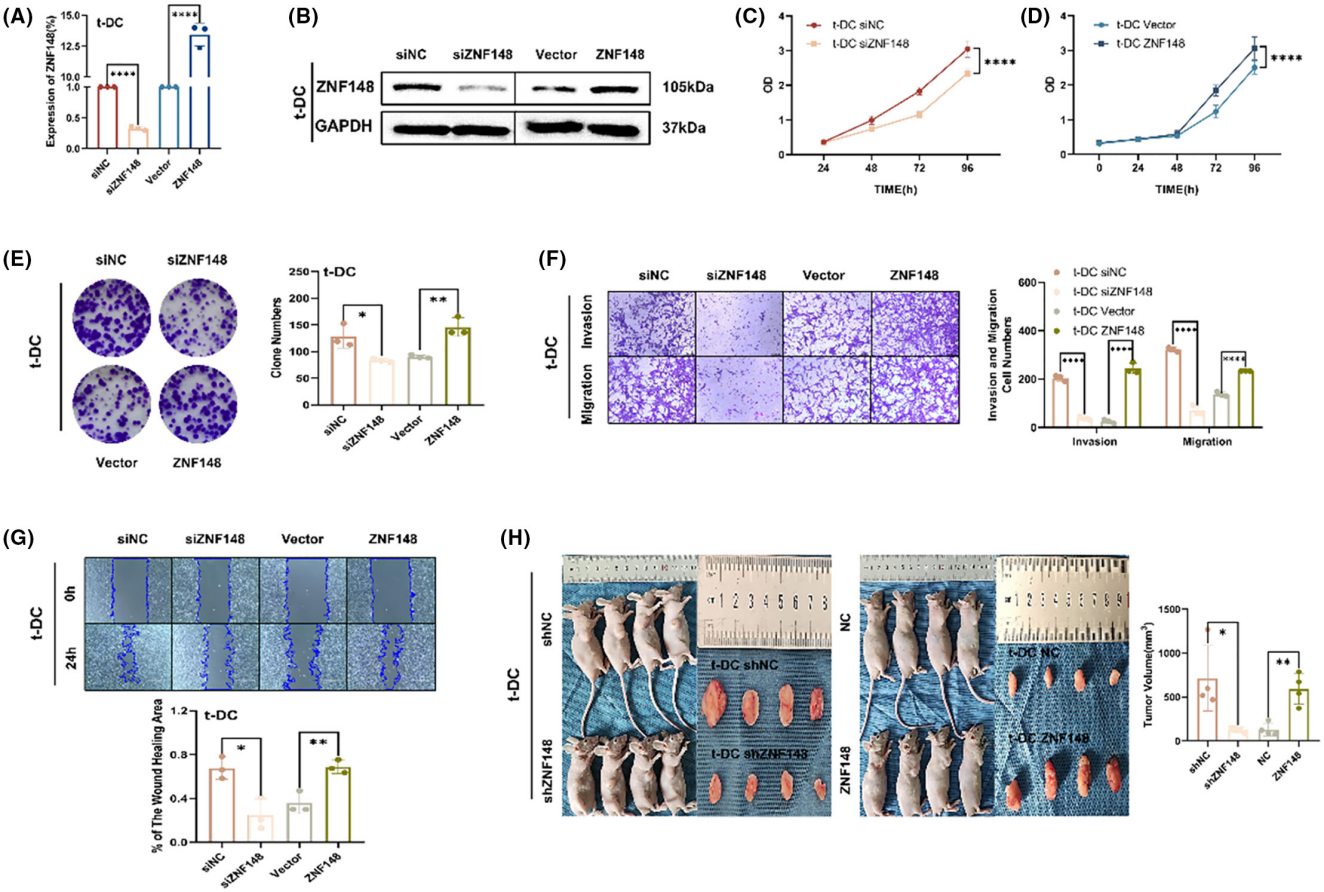
Knockdown or upregulation of the transcription factor ZNF148 suppressed or promoted the malignant phenotype of t‐DCs, respectively. (A, B) The transfection efficiency was verified by qRT‐PCR and western blot. (C–E) CCK8 and clone formation assay of t‐DCs transfected with si‐NC, si‐ZNF148, Vector, or ZNF148‐overexpression plasmids. (F, G) Transwell and wound healing assay of t‐DCs transfected with si‐NC, si‐ZNF148, Vector, or ZNF148‐overexpression plasmids. (H) Tumorigenicity assay in nude mice after subcutaneous inoculation of shZNF148 or ZNF148 t‐DCs. Data are expressed as mean ± SD, *p < 0.05, **p < 0.01, ***p < 0.001.

T‐DCs in which ZNF148 was downregulated or overexpressed were constructed by siRNA and plasmid transfection, respectively. The transfection efficiency was verified by qRT–PCR and western blotting (Figure 4A,B). Both CCK8 and colony formation assays indicated that ZNF148 silencing impaired the proliferation of t‐DCs, whereas ZNF148 overexpression accelerated cell growth (Figure 4C–E). Transwell assay showed that ZNF148 knockdown resulted in a significant decrease in the invasion of t‐DCs, while ZNF148 overexpression exerted the opposite effects (Figure 4F). Downregulation of ZNF148 also markedly decreased the migration of t‐DCs, as shown in the wound healing assay, while ZNF148 overexpression exerted the opposite effects (Figure 4F,G). The tumorigenicity assay indicated an obvious decrease in subcutaneous tumor volumes when ZNF148 was knocked down in t‐DCs compared with the control group, while ZNF148 overexpression exerted the opposite effects (Figure 4H).

### 3.6 PTX3 upregulation promoted the malignant phenotype of t‐DCs

**FIGURE 5:**
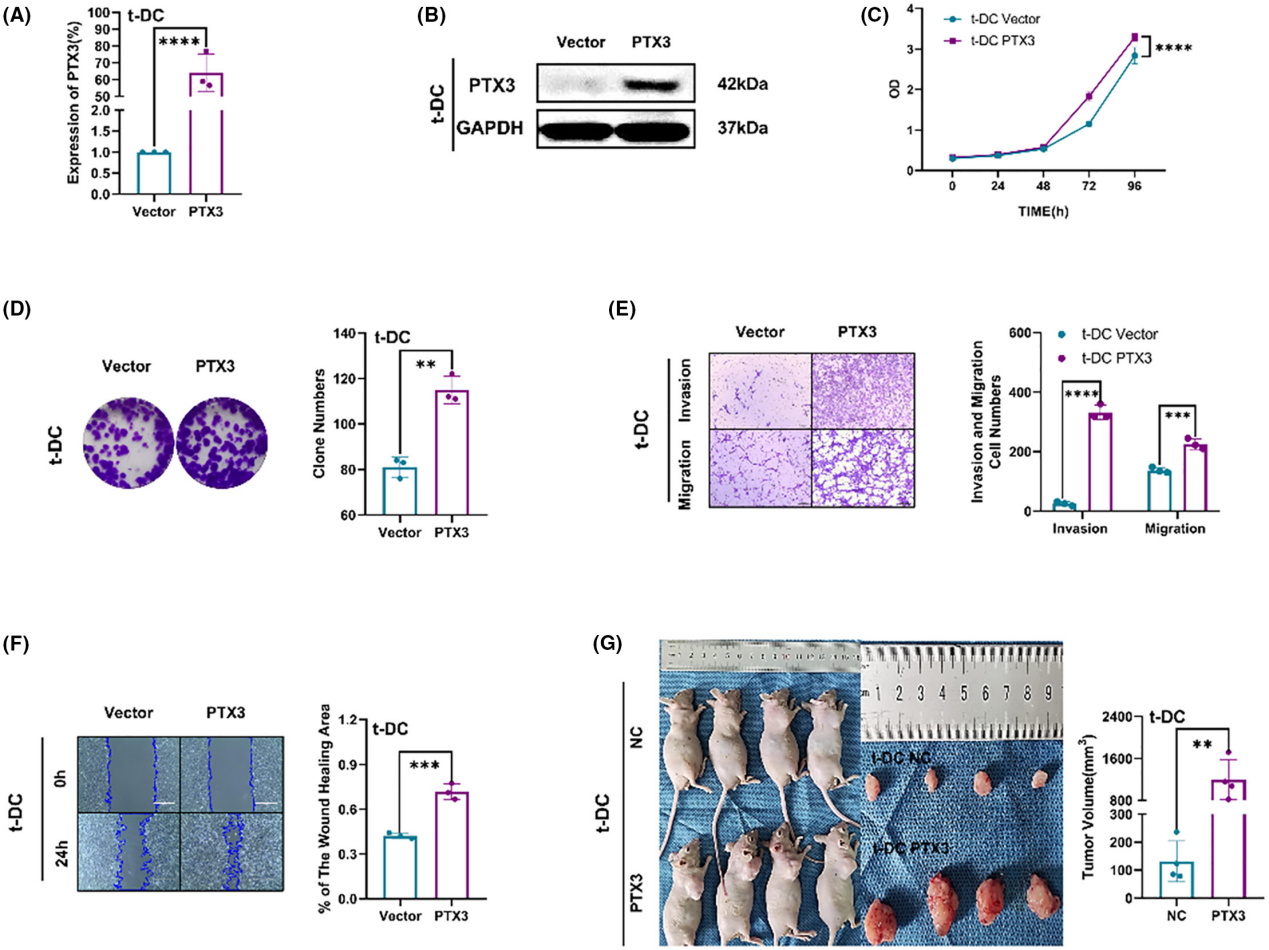
PTX3 upregulation promoted the malignant phenotype of t‐DCs. (A, B) The transfection efficiency was verified by qRT‐PCR and western blot. (C) CCK8 assay on t‐DCs with PTX3 upregulation. (D) Clone formation assay on t‐DCs with PTX3 upregulation. (E, F) Transwell and wound healing assay of t‐DCs with PTX3 upregulation. (G) Tumorigenicity assay performed in nude mice by subcutaneous inoculation of t‐DCs with PTX3 upregulation. Data are expressed as mean ± SD, *p < 0.05, **p < 0.01, ***p < 0.001.

To validate the proliferation‐promoting effect of PTX3 on t‐DCs, overexpression of PTX3 in t‐DCs was achieved via transfection of the PTX3 vector, and the transfection efficiency was verified by qRT–PCR and western blotting (Figure 5A,B). CCK8 and colony formation assays indicated that the overexpression of PTX3 promoted the proliferation of t‐DCs (Figure 5C,D). Transwell assays showed that the overexpression of PTX3 promoted the invasion and migration of t‐DCs, as shown in the wound healing assay (Figure 5E,F). The tumorigenicity assay indicated an obvious increase in the tumor volume of mice that were subcutaneously implanted with PTX3‐overexpressing t‐DCs compared with the control group (Figure 5G).

### 3.7 PTX3 is involved in ZNF148‐mediated malignant phenotypes

**FIGURE 6:**
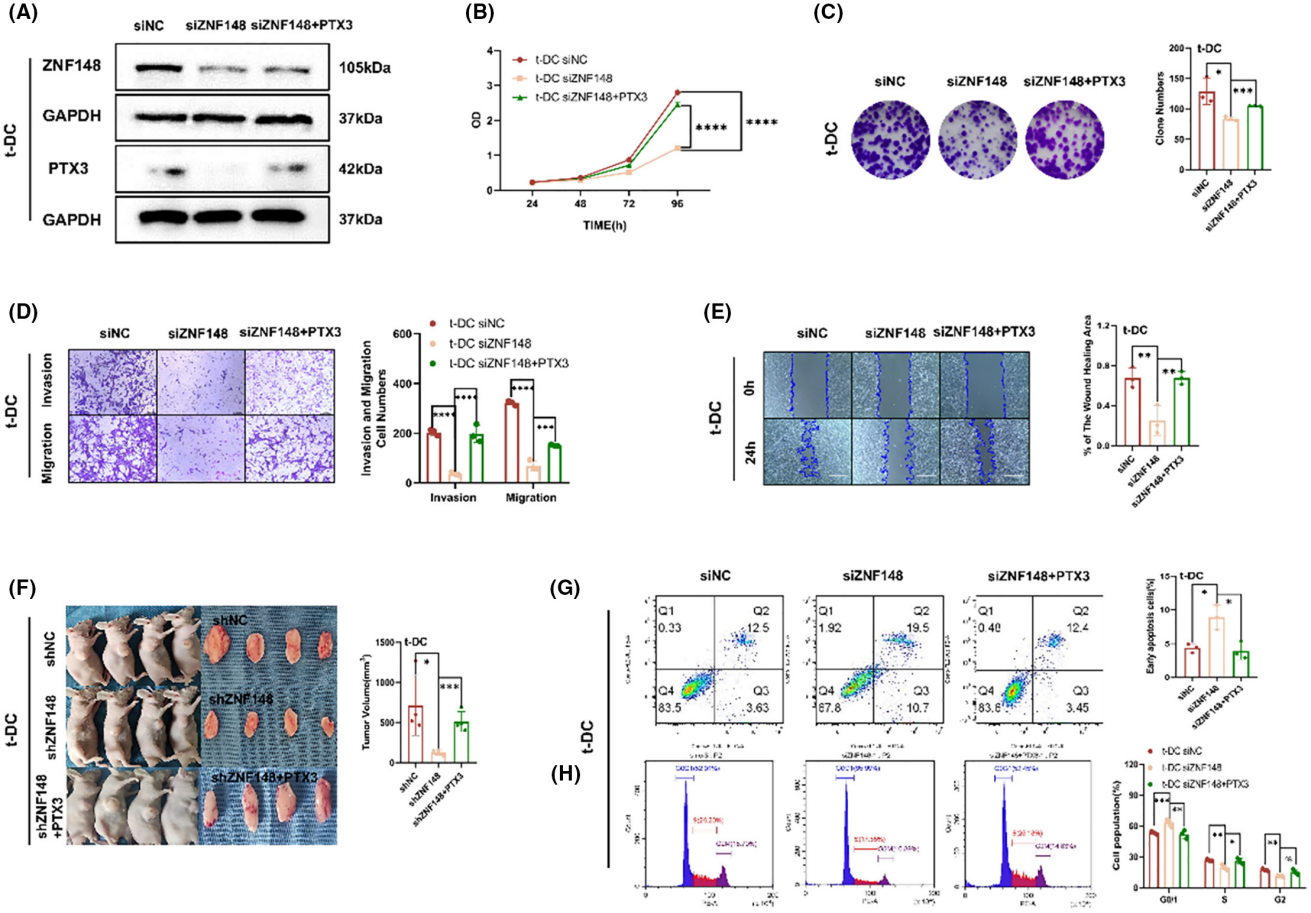
PTX3 is involved in ZNF148‐mediated malignant phenotypes. (A) Levels of PTX3 in t‐DCs transfected with si‐NC, si‐ZNF148, or si‐ZNF148 with PTX3‐overexpression plasmids by western blot. (B, C) CCK8 and clone formation assay of t‐DCs transfected with si‐NC, si‐ZNF148, or si‐ZNF148 with PTX3‐overexpression plasmids. (D, E) Transwell and wound healing assay of t‐DCs transfected with si‐NC, si‐ZNF148, or si‐ZNF148 with PTX3‐overexpression plasmids. (F) Tumorigenicity assay performed in nude mice by subcutaneous inoculation of t‐DCs with sh‐NC, sh‐ZNF148, or sh‐ZNF148 with PTX3‐overexpression. (G) Flow cytometry on cell apoptosis of t‐DCs transfected with si‐NC, si‐ZNF148, or si‐ZNF148 with PTX3‐overexpression plasmids. (H) Cell cycle assay of t‐DCs transfected with si‐NC, si‐ZNF148, or si‐ZNF148 with PTX3‐overexpression plasmids. Data are expressed as mean ± SD, *p < 0.05, **p < 0.01, ***p < 0.001.

To investigate the role of PTX3 in ZNF148‐mediated t‐DC development, t‐DCs were transfected with NC, si‐ZNF148 alone, or si‐ZNF148 with PTX3 overexpression plasmids. The levels of PTX3 in these cells were measured by western blotting. The results showed that si‐ZNF148 decreased the levels of PTX3, while ZNF148 overexpression reversed this effect (Figure 6A). Moreover, CCK8 and colony formation assays showed that PTX3 overexpression abolished the si‐ZNF148‐mediated inhibition of proliferation (Figure 6B,C). Similarly, the transwell and wound healing assays indicated that PTX3 overexpression reversed the si‐ZNF148‐mediated suppression of migration and invasion in t‐DCs (Figure 6D,E). The tumorigenicity assay indicated an obvious decrease in subcutaneous tumor volumes when ZNF148 was knocked down in t‐DCs, and this effect could be partially reversed by the simultaneous upregulation of PTX3 (Figure 6F). Flow cytometry analysis showed that there was also an increase in the fraction of early apoptotic cells after the knockdown of ZNF148 compared with the control cells, and this effect could be partially reversed by the simultaneous upregulation of PTX3 (Figure 6G). Cell cycle analysis revealed that the downregulation of ZNF148 led to cell cycle arrest in the G_0_–G_1_ phase in t‐DCs and this effect could also be partially reversed by the simultaneous upregulation of PTX3 (Figure 6H). These data indicated that ZNF148 promoted the malignant phenotype of t‐DCs by regulating PTX3.

### 3.8 The ZNF148/PTX3 axis downregulated adhesion and costimulatory molecule expression in t‐DCs

**FIGURE 7:**
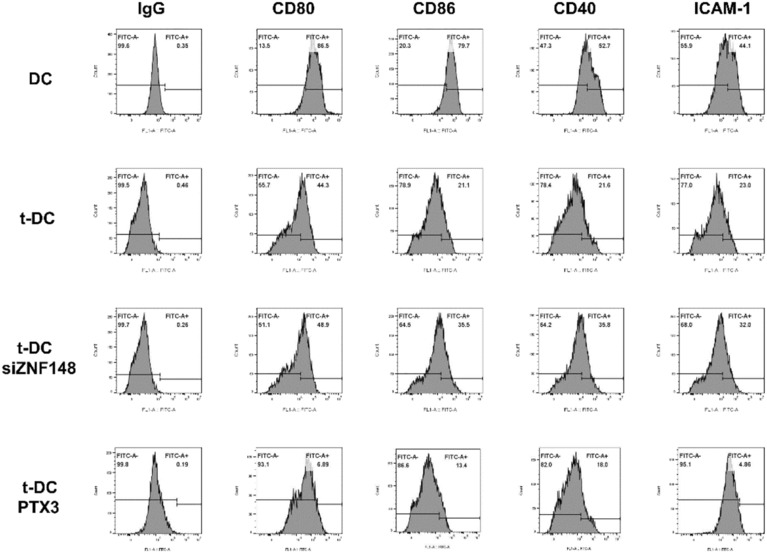
ZNF148/PTX3 axis downregulated expression of adhesion and costimulatory molecules of t‐DCs.

To investigate the effect of the ZNF148/PTX3 axis on t‐DC phenotype, the expression of the costimulatory molecules CD80, CD86, and CD40 and the adhesion molecule ICAM‐1 before and after the ZNF148/PTX3 axis was altered was examined by flow cytometry. Compared with that in normal DCs, the expression of the costimulatory molecules CD80, CD86, and CD40 and the adhesion molecule ICAM‐1 in t‐DCs decreased significantly, suggesting that t‐DCs behaved like immune‐tolerant DCs to some extent.^7^, ^8^ However, the knockdown of the transcription factor ZNF148 increased the expression of the costimulatory molecules CD80, CD86, and CD40 and the adhesion molecule ICAM‐1. Overexpression of PTX3 further decreased the expression of the costimulatory molecules CD80, CD86, and CD40 and the adhesion molecule ICAM‐1 in t‐DCs (Figure 7), which indicated the important regulatory role of the ZNF148/PTX3 axis in promoting the tolerogenic phenotype of DCs.

### 3.9 The transcription factor ZNF148 regulated PTX3 expression by directly binding to its promoter region

**FIGURE 8:**
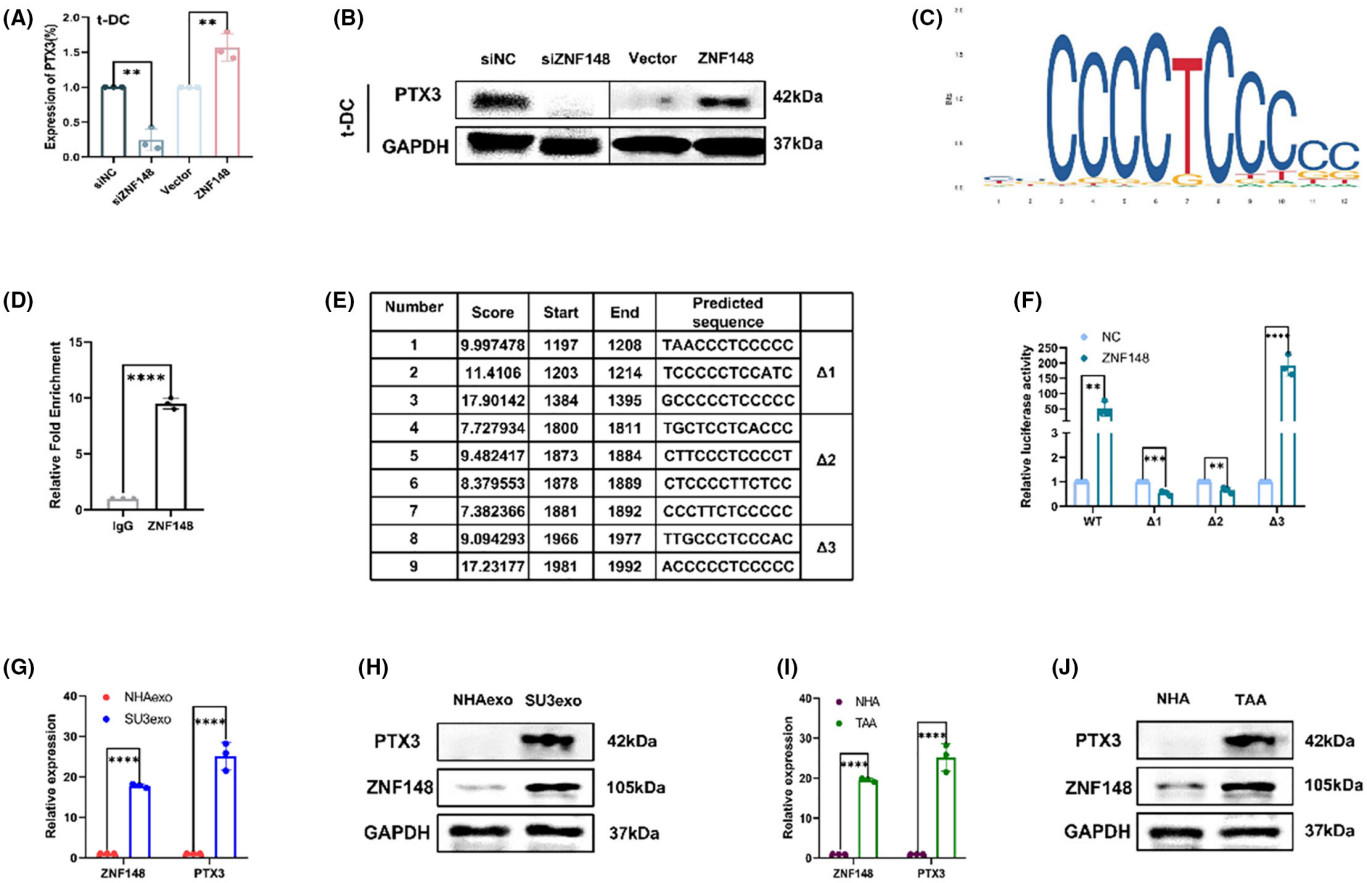
The transcription factor ZNF148 regulated PTX3 expression by directly binding to its promoter region. (A, B) Levels of PTX3 in t‐DCs transfected with si‐NC, si‐ZNF148, Vector, or ZNF148‐overexpression plasmids by qRT‐PCR and western blot. (C) ZNF148 binding site in PTX3 promoter region predicted by UCSC. (D) ChIP‐qPCR assay indicating binding of ZNF148 with PTX3 promoter. (E) Oligonucleotide sequences 1 to 9 represent the predicted ZNF148 binding sites by JASPAR. (F) Dual‐luciferase reporter assay indicating binding of PTX3 with ZNF148. (G) Quantitative RT‐PCR on ZNF148 and PTX3 expression in SU3 cells‐derived exosomes. (H) Western blot on ZNF148 and PTX3 expression in SU3 cells‐derived exosomes. (I) Quantitative RT‐PCR on ZNF148 and PTX3 expression in TAAs. (J) Western blot on ZNF148 and PTX3 expression in TAAs. Data are expressed as mean ± SD, *p < 0.05, **p < 0.01, ***p < 0.001.

Knockdown of the transcription factor ZNF148 directly reduced the expression of PTX3, and overexpression of the transcription factor ZNF148 directly increased the expression of PTX3 (Figure 8A,B). Analysis of the UCSC database revealed that ZNF148‐binding sites are located in the 5′‐untranslated region (UTR) of PTX3, comprising a 2000‐bp sequence upstream of the transcription start site (Figure 8C). ChIP‐qPCR assay showed that transcription factor ZNF148 was directly bound to the PTX3 promoter (Figure 8D). According to the JASPAR database, nine candidate sequences in the PTX3 promoter region were predicted to serve as potential ZNF148 binding sites. Therefore, we constructed a vector encoding the full‐length PTX3 promoter (pGL3‐PTX3) as well as vectors encoding three PTX3 truncated promoter constructs that spanned the three central high‐scoring sequences (i.e., sequences with relatively strong binding potential: pGL3‐PTX3‐Δ1, pGL3‐PTX3‐Δ2, and pGL3‐PTX3‐Δ3; Figure 8E). A dual‐luciferase reporter assay was performed by cotransfecting t‐DCs with ZNF148 and pGL3‐PTX3 or three truncated fragments (Δ1, Δ2, or Δ3). Transformed DCs cotransfected with ZNF148 and PGL3‐PTX3‐Δ3 exhibited the highest luciferase activity (Figure 8F). This indicated that ZNF148 is bound to the 1196–1992 region of the PTX3 promoter.

### 3.10 Indirect verification of the role of ZNF148/PTX3 axis on t‐DCs in GSCs‐microenvironment

Quantitative RT‐PCR and western blotting were performed to analyze ZNF148 and PTX3 content in SU3 cells derived exosomes, which disclosed that both ZNF148 and PTX3 were upregulated in SU3 exosomes at transcriptional as well as protein levels, compared with normal human astrocytes (NHAs) exosomes (Figure 8G,H). Besides, intracellular expression of ZNF148 and PTX3 increased obviously in tumor‐associated astrocytes (TAAs) at both transcriptional and protein levels, compared with NHAs (Figure 8I,J).

## 4 DISCUSSION

Most studies have attributed the treatment resistance and high recurrence of gliomas to GSCs^36^, ^37^, ^38^ based on their infinite self‐renewal capacity, persistent proliferation, and strong ability to remodel the TME.^39^, ^40^, ^41^ Various stromal cells in the TME actively interact with GSCs and play vital roles in GSC‐induced tumor progression. Various glioma biological processes are largely affected by the TME,^42^ and targeting the TME has been considered a potential treatment strategy in recent years.^43^ The unique properties of TME stromal cells, which have emerged as critical regulators of cancer tissue remodeling, indicate a specific framework that needs to be taken into consideration in the design of TME‐targeted interventions.^9^, ^44^

DCs are bone marrow‐derived, specialized antigen‐presenting cells (APCs) that function within the immune system to bridge innate and adaptive immunity.^45^ As dominant APCs, DCs capture tumor antigens and present them to antigen‐specific T cells via MHC glycoproteins, thereby activating naive T cells and initiating specific immune responses.^46^ Upon pathogen sensing, populations of DCs in peripheral tissues become activated and migrate to the T‐cell areas of draining lymph nodes (LNs), where they stimulate antigen‐specific T‐cell responses to initiate immunity or tolerance.^47^ These cells are termed migratory DCs.^48^ In addition to their “educator” role, DCs can secrete cytokines and growth factors to regulate the tissue environment.^49^ Immature DC cells exposed to a hypoxic microenvironment express high levels of HIF‐1α and upregulate B‐cell lymphoma 2 (Bcl‐2)/adenovirus E1B 19‐kd interacting protein 3 (BNIP3), which mediates DC Programmed cell death.^50^ Additionally, hypoxia reduces the surface expression of DC differentiation and maturation markers, including MHC‐II and costimulatory molecules (CD40, CD80, and CD86).^51^ Glioma cells suppress the expression of MHC‐II through TGF‐β1.^52^ As interest in the use of immune checkpoint inhibitors for cancer therapy continues to expand, DC vaccines are likewise gaining significant clinical attention as an alternative strategy to stimulate T‐cell responses. Preconditioning of the vaccination site with an inflammatory stimulus (e.g., tetanus toxoid) was found to significantly increase DC homing to nearby draining LNs, leading to prolonged progression‐free and overall survival of glioblastoma patients; these results demonstrated the potential of DC vaccines to exert antitumor effects on glioblastoma. DC vaccinations, such as DC‐Vax‐L, have yielded promising results and are being evaluated in advanced clinical trials (newly diagnosed glioblastoma, NCT00045968, phase III).^53^ This implies that DC‐based immunotherapy may have great potential for the treatment of gliomas.

We previously reported the malignant transformation of oligodendrocytes, macrophages, MSCs, and fibroblasts induced by GSCs in a dual‐color tracing orthotropic GSC model, and revealed that differentiated non‐GSCs glioma cells cannot induce malignant transformation of these cells.^20^, ^54^ The current study aimed to investigate the crosstalk between GSCs and DCs. Based on the double‐fluorescence tracing platform, interactions between GSCs labeled with RFP and DCs labeled with GFP were visible. After approximately 10 days of co‐culture, a few green cells (DCs) were found to undergo active proliferation. As coculture continued, these cells became increasingly prevalent. After monoclonal with a micropipetting technique under a fluorescence microscope, t‐DCs were found to retain the expression of DC surface markers and to behave like transformed cells with high proliferation, strong invasion and migration abilities, and definite tumorigenicity.

PTX3, which is also called tumor necrosis factor (TNF)‐inducible gene 14 protein (TSG‐14), is a member of the superfamily of acute‐phase proteins.^55^ PTX3 activates JNK signaling and regulates the epithelial‐to‐mesenchymal transition,^56^ which is always accompanied by tumor metastasis.^57^ Increasing amounts of evidence have focused on the potential relationship between PTX3 and various malignancies. Increased plasma levels of PTX3 are associated with a poor prognosis in colorectal carcinoma patients.^58^ Overexpression of PTX3 is related to poor prognosis in lung cancer patients via a local inflammatory response.^59^ Higher levels of PTX3 were observed in glioblastomas, whereas its expression in low‐grade gliomas and normal astrocytes is very low or null,^60^ and higher levels of PTX3 are associated with a high degree of malignancy and shorter patient survival.^61^ Both stromal cells (endothelium and fibroblasts) and immune cells (macrophages, neutrophils, and DCs) in the GBM microenvironment are known to produce PTX3.^62^ PTX3 is an important component of the GBM microenvironment that is produced by both tumor cells and infiltrating CD68‐positive macrophages, and local PTX3 levels correlate with glioma grade and malignancy.^60^ ZNF148 is a Kruppel‐type zinc finger family protein that binds to GC‐rich sequences in various gene promoters.^63^, ^64^ ZNF148 can act as a transcriptional regulator to activate or repress gene expression.^65^ Previous studies indicate that ZNF148 potentially acts as a tumor‐promoting factor in many tumors,^64^, ^66^, ^67^, ^68^, ^69^, ^70^ so the implications of ZNF148 in tumor development in the context of genetics are worthy of further investigation. Additionally, our results showed higher expression of ZNF148 and higher expression of PTX3 in highly invasive malignant t‐DCs. Upregulated ZNF148 expression resulted in increased cell growth and invasion by upregulating PTX3, which further confirmed that the role of DCs can be modulated by GSC‐mediated remodeling of the immune microenvironment. However, the molecules released by GSCs that induce the transformation of DCs through activating the ZNF148/PTX3 axis still need further investigation.

Overall, our study demonstrated that DCs can be induced to undergo malignant transformation after being cocultured with GSCs in vitro, and the ZNF148/PTX3 axis plays an important role in the malignant transformation of DCs. Our findings provide strong evidence that the transformation of DCs can occur in the highly immunosuppressive microenvironment that is generated by GSCs. Therefore, the development of novel DC‐based immunotherapies for the treatment of gliomas requires caution, and this area still needs further investigation. However, there is an existing common problem associated with most glioma research, namely, the lack of complete immunity in nude mice, which limited the interpretation of our experimental data to some extent. Our experimental design can be improved if applying more powerful in vivo characterization methods to further elucidate the role of transformed DCs in GSCs‐microenvironment. Recent studies have reported that the deficiency of nude mice models can be improved by adopting several brain imaging modalities,^71^, ^72^ which have been developed that are particularly appropriate for in vivo glioma studies. Especially, molecular magnetic resonance imaging (MRI) to visualize biological processes at the molecular level in nude mouse models, helps to enhance feasibility and accuracy in future studies.^71^

## AUTHORS' CONTRIBUTIONS

JD conceived the study. SC, LL, and DL‐W performed experiments. YD‐L and SW‐L participated in animal experiments. JQ‐Y and SL‐H performed the statistical analysis. SC and ZP‐X drafted the paper. JD supervised the study. All authors have read and approved the final manuscript.

## FUNDING INFORMATION

This study was supported by Jiangsu Province Key Research and Development Program: Social Development Project (BE2021653), Natural Science Foundation of Jiangsu Province (BK20201172), and Key Program of Health Commission of Jiangsu Province (ZBD2020016).

## CONFLICT OF INTEREST STATEMENT

This manuscript has not been published elsewhere and is not under consideration by another journal. We have read and understood your journal's policies, there are no conflicts of interest to declare.

## DATE AVAILABILITY STATEMENT

The raw data supporting the conclusions of this article will be made available by the authors.

## Supporting information


Figure S1

Appendix S1
