# Supplementary figures and images for: Upregulation of the ZNF148/PTX3 axis promotes malignant transformation of dendritic cells in glioma stem‐like cells microenvironment
Source: CNS Neurosci Ther. 2023 Apr 17;29(9):2690–704. doi: 10.1111/cns.14213 (PMC10401131; doi:10.1111/cns.14213)

**A**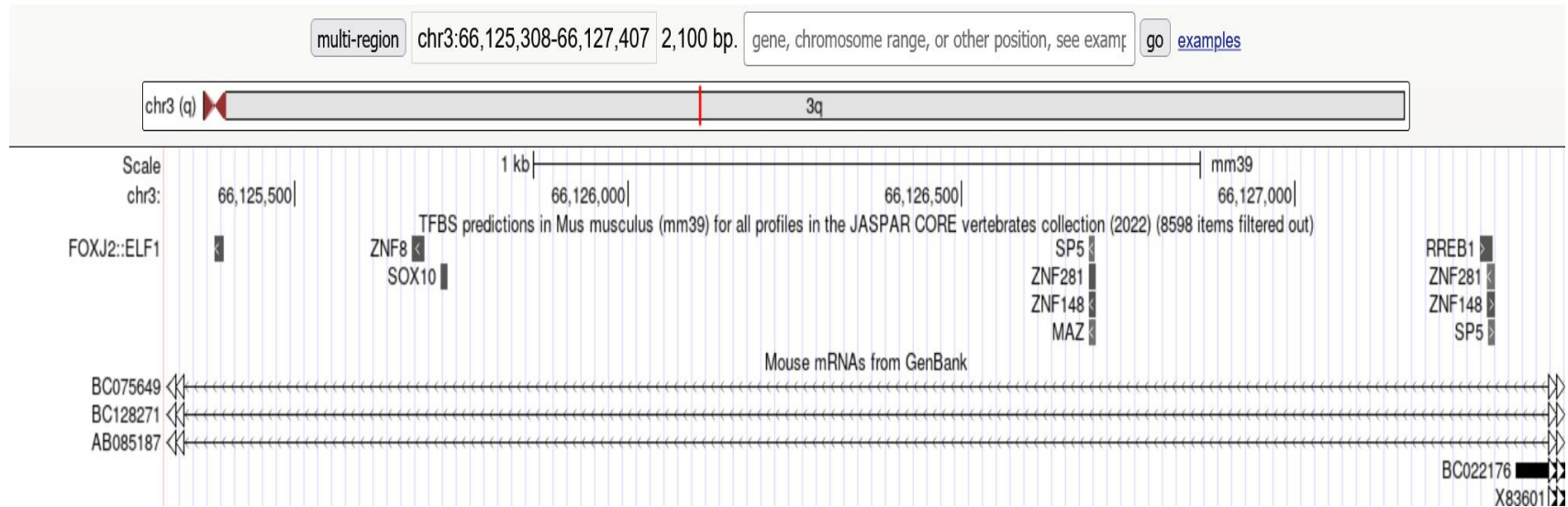**B**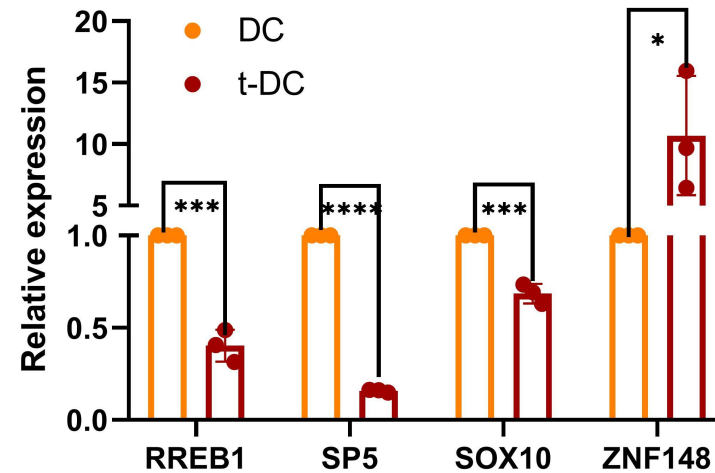

**Figure S1** Expression of ZNF148 in t-DCs

Supplement: Supplementary file 1 — Figure S1 [file CNS-29-2690-s002.pdf]
