# Supplementary material for: Upregulation of the ZNF148/PTX3 axis promotes malignant transformation of dendritic cells in glioma stem‐like cells microenvironment
Source: CNS Neurosci Ther. 2023 Apr 17;29(9):2690–704. doi: 10.1111/cns.14213 (PMC10401131; doi:10.1111/cns.14213)

A Original full unedited WB gel/blot for ZNF148 expression in Figure 3G, 4B, 6A

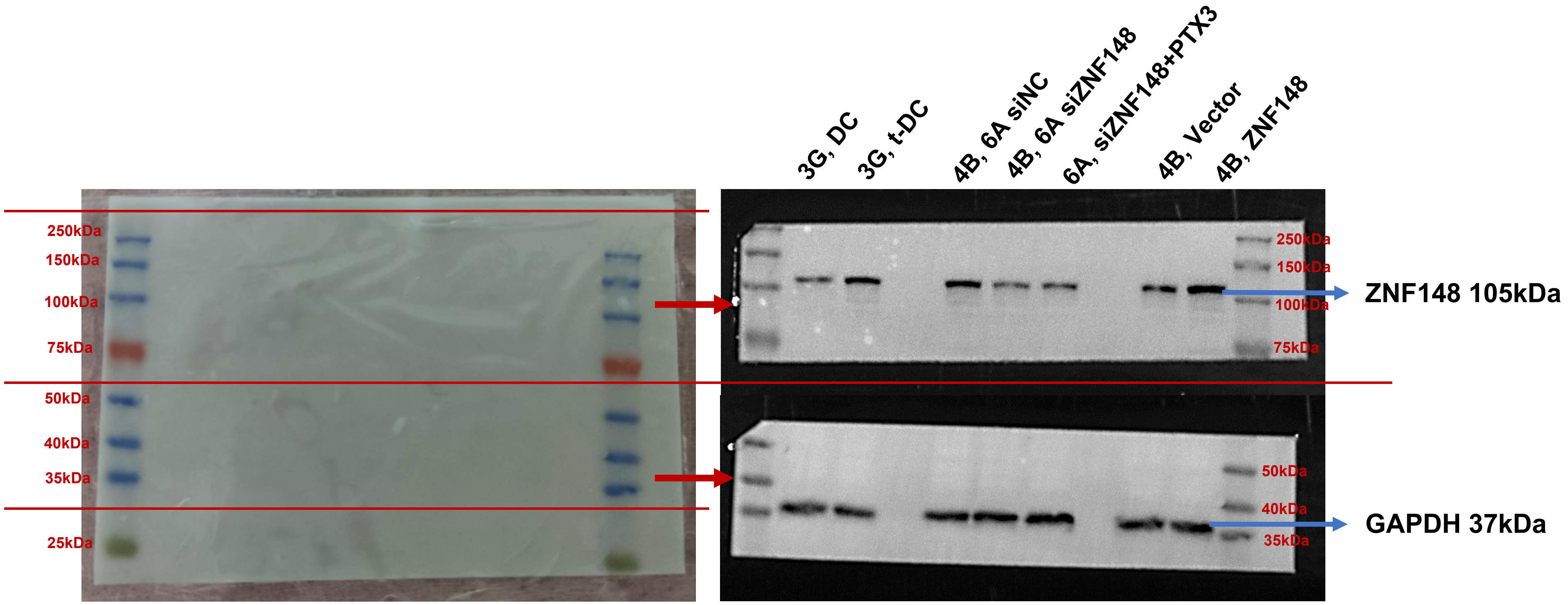

B Formal Figure 3G, 4B, 6A in the manuscript

Figure 3G:

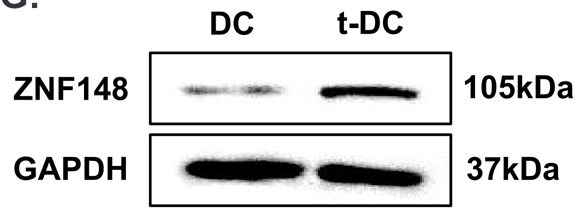

Figure 6A:

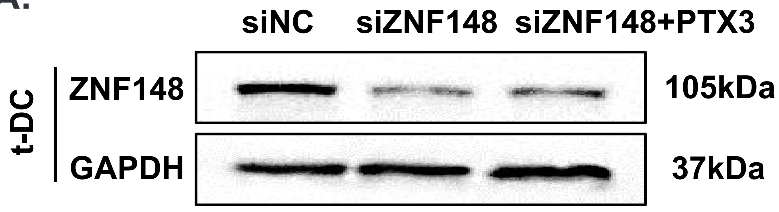

Figure 4B:

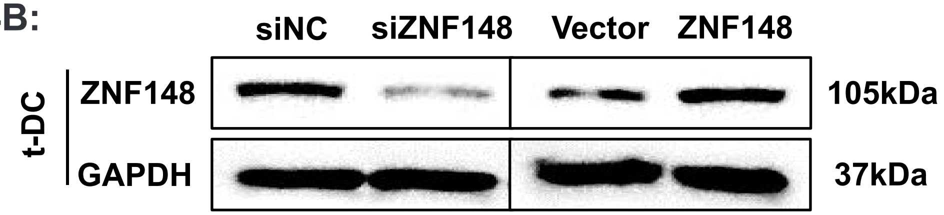

A Original full unedited WB gel/blot for PTX3 expression in Figure 3G, 8B

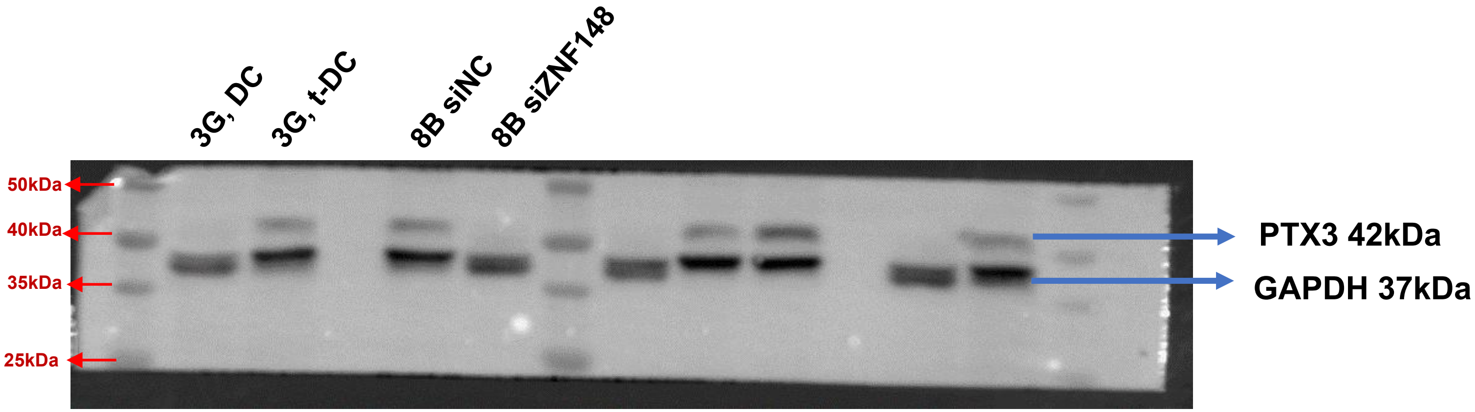

B Formal Figure 3G, 8B in the manuscript

Figure 3G:

Figure 8B:

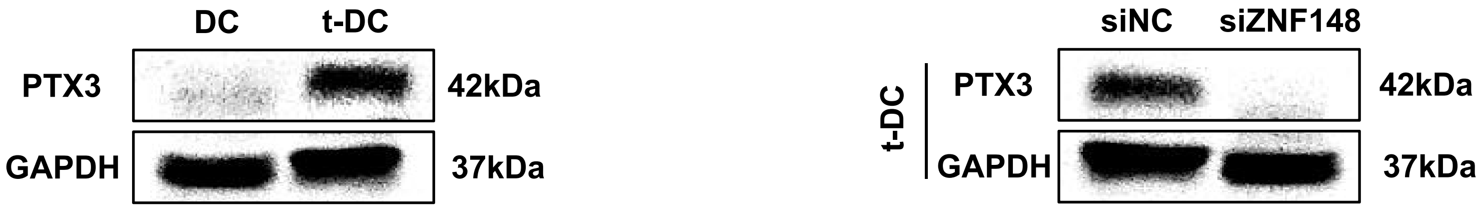

A Original full unedited WB gel/blot for PTX3 expression in Figure 6A

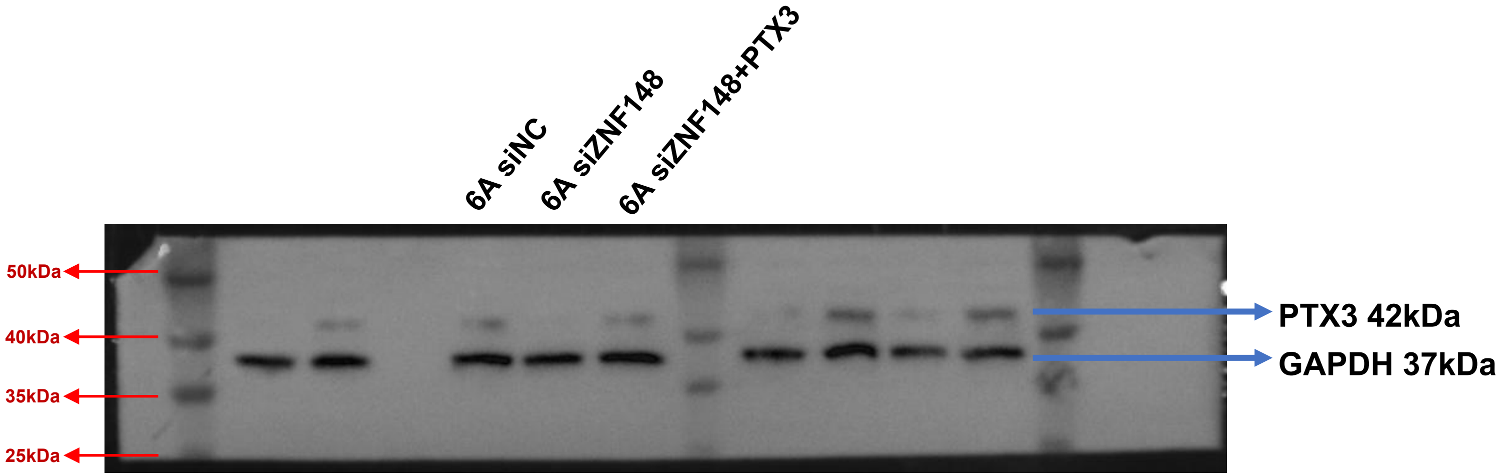

B Formal Figure 6A in the manuscript

Figure 6A:

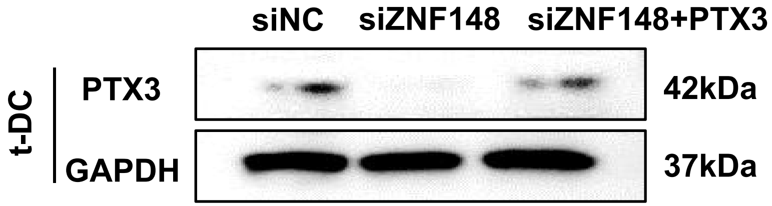

A Original full unedited WB gel/blot for PTX3 expression in Figure 5B, 8B

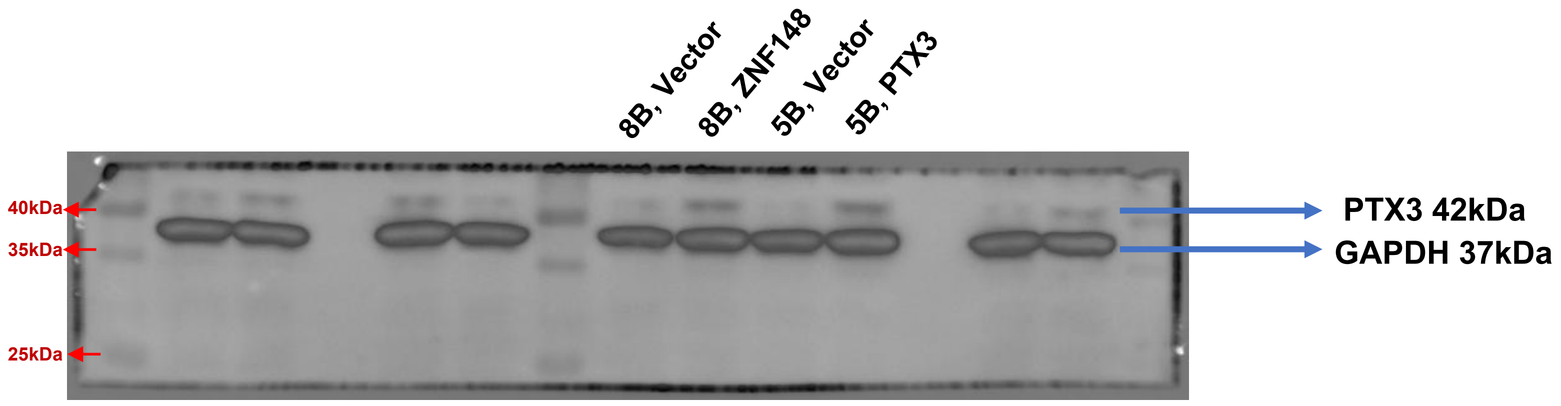

B Formal Figure 5B, 8B in the manuscript

Figure 5B:

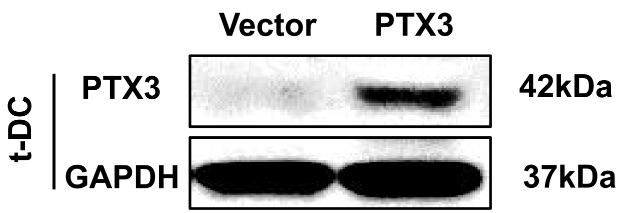

Figure 8B:

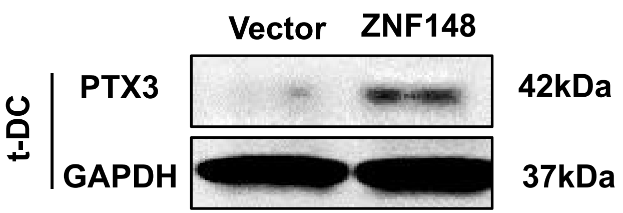

Supplement: Supplementary file 2 — Appendix S1 [file CNS-29-2690-s001.pdf]
